# An Interactive Astronaut-Robot System with Gesture Control

**DOI:** 10.1155/2016/7845102

**Published:** 2016-04-11

**Authors:** Jinguo Liu, Yifan Luo, Zhaojie Ju

**Affiliations:** ^1^State Key Laboratory of Robotics, Shenyang Institute of Automation, Chinese Academy of Sciences, Shenyang 110016, China; ^2^University of Chinese Academy of Sciences, Beijing 100864, China; ^3^School of Computing, University of Portsmouth, Portsmouth, Hampshire PO1 3HE, UK

## Abstract

Human-robot interaction (HRI) plays an important role in future planetary exploration mission, where astronauts with extravehicular activities (EVA) have to communicate with robot assistants by speech-type or gesture-type user interfaces embedded in their space suits. This paper presents an interactive astronaut-robot system integrating a data-glove with a space suit for the astronaut to use hand gestures to control a snake-like robot. Support vector machine (SVM) is employed to recognize hand gestures and particle swarm optimization (PSO) algorithm is used to optimize the parameters of SVM to further improve its recognition accuracy. Various hand gestures from American Sign Language (ASL) have been selected and used to test and validate the performance of the proposed system.

## 1. Introduction

When astronauts conduct EVA missions on the surface of other planets, they generally need to collaborate with some agents or some systems to complete the missions smoothly and efficiently. Reducing the crew workload is a primary concern, particularly during EVA. The robot's autonomy can make the robot finish some tasks independently and allow the robot to complete certain tasks with little crew's attention. The robot used in the space exploration always has a high level of autonomy (LOA). However, in current real operations, a human operator has a better insight in the task completion than the robot system. Autonomous systems are not yet as efficient as humans in modeling the richness of interactions and balancing the trade-off between the various crewmembers and their mission requests. Therefore, astronauts must interact with the robot at various levels, from high level goal commands to detailed activity sequences and then to direct teleportation, to cope with the full spectrum of situations expected. This creates significant challenges with regard to communication, human-robot interface, and human-understandable state representation.

As for the HRI problem, considerable effort has been made to the development of intelligent and natural interfaces between users and computer systems, and HRI has been developed by leaps and bounds [1–6]. Now there are many mature ways of HRI; among those ways, voice recognition and gesture recognition are two major developing directions. Speech recognition system now is developing towards two important directions: one is the large vocabulary continuous speech recognition system and the other is the application of miniaturization, portable audio products. The large vocabulary and continuous speech recognition system is now generally based on one or more PCs. The portable processing chip for recognition usually has limitations in computing speed and storage capacity. In planetary exploration missions, these limitations indicate that there is still a long way to go to apply speech recognition in this area. Hand gestures, which have been addressed in the sign language for the deaf people for many years, can represent rich language and have also attracted a lot of attention. Gesture recognition is a technology often used in HRI applications, and there are lots of methods for hand gesture recognition, such as the methods based on image recognition, curvature, and surface electromyography (EMG) signal.

**Figure 1:**
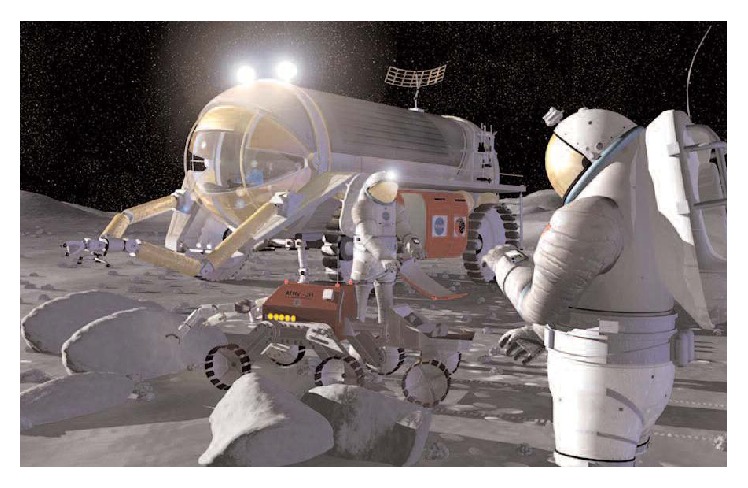
Schematic diagram of astronauts collaborating with agent [3].

This paper proposes a way of using hand gestures of astronauts to intervene in the autonomy of the agent. An example of astronauts cooperating with agents to complete a mission is shown in Figure 1. Though recent image processing techniques have achieved a fascinating development [2], they are not suitable for the space applications, because the clumsy suit may bring some of the most difficult problems in the field of machine vision [7]. For surface EMG signals, there is a large gap in the space suits and the atmospheric pressure inside spacesuit is only 40 percent of the standard atmosphere, so whether the EMG signals in this case change or not is unknown.

Increasing numbers of industrial and service robots [8, 9] have focused on designing the HRI technology in order to increase robot efficiency and effectiveness. HRI refers to a process of conveying operators' intentions and interpreting the sequence of robot motions and working requirements in task descriptions. The complement of HRI through the application of suitable interaction methods and interfaces has been an essential factor as well as a challenge in the robot industry. Recent development of robotics has introduced haptic interaction, through which the users can feel both virtual and real environments, such as in teleoperations and telesurgeries [10]. There have been many works providing technical and theoretical support for HRI to be more efficient and suitable. Now commonly used methods include multimodal interaction, teaching model, virtual reality, and augmented reality.

Nowadays, the space activity is still in the early stage, and the technology needs further improvement. In the near future, with the development of aerospace technology, the astronauts will not be limited to the technical personnel; other people, such as engineers, physicists, biologists, surgeons, and even philosophers, also have the opportunities to become astronauts in the space exploration and carry out relevant scientific experiments. Therefore, the individual agent or multiagent system, which collaborates with astronauts, requires a higher LOA and friendly HRI. Making HRI more effective, efficient, and natural is crucial to the success of sustained space exploration. In particular, we assume that humans and robots must be able to (1) communicate clearly about their goals, abilities, plans, and achievements; (2) collaborate to solve problems, especially when situations exceed autonomous capabilities; and (3) interact via multiple modalities (dialogue, gestures, etc.), both locally and remotely. To achieve these goals, a number of HRI challenges must be addressed.

Using gestures to convey information has become an important part of human computer interaction [4–7]. Hand gesture recognition is widely used in many applications, such as computer games, machinery control (e.g., crane), and household electrical appliance remote control. Hand gesture analysis can be divided into three main approaches, namely, glove-based methods, vision-based methods, and methods for drawing gestures [5]. For approaches based on the data-glove, the relative position of a finger is captured by an additional sensor, which is normally a magnetic or acoustic sensor attached to a glove. A lookup table software toolkit is usually provided for hand gesture recognition [7]. The second way is based on the image processing, which is stricter with the image background, and thus it is not suitable for applications in a complex working environment [6]. The third method involves the analysis of gesture drawing [5], using a stylus as an input device. This method is often used for identifying written words, which has problems of reliability, accuracy, and electromagnetic interference noise.

The paper is organized as follows. In Section 2, the interactive astronaut-robot system is introduced in detail, including the system devices, the overall plan and the main functions, and the snake-like robot. In Section 3, we introduce the application of SVM and PSO for the hand gesture recognition. In Section 4, we designed two experiments to verify the reliability and robustness of the proposed system. Conclusions and future work are discussed in Section 5.

## 2. Interactive Astronaut-Robot System

The system integrates bending sensors in a glove to capture the bending angles of all the fingers. Then the finger angles are classified through the model trained by the SVM, and corresponding instructions generated control the snake-like robot, so that the snake-like robot can assist astronauts to complete the mission. The main components include bending sensor system, STM32 controller, wireless communication module, and the modular snake robot composed with servos. The main parameters of each device are shown in Table 1.

### 2.1. The Control System

**Figure 2:**
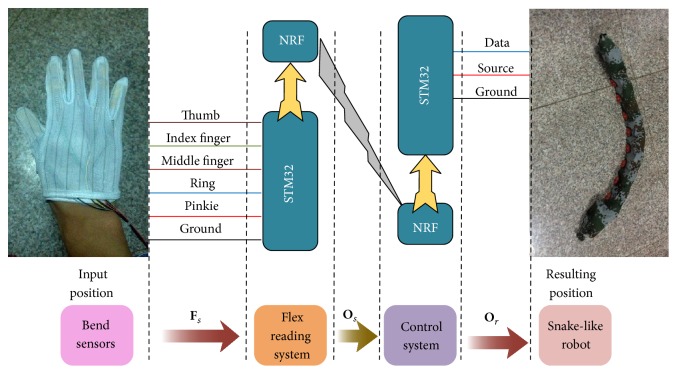
Outline of the control system.

The main function of this control system is designed to achieve the modular robot moving with the planned movement according to the instructions from the gesture recognition system. Detailed implementation is shown in Figure 2. After the controller gets the signal **F**_*s*_ from the bend sensors mounted on the glove, the signal goes through a filter and a normalization preprocessing stage, and **O**_*s*_ is sent to the controller mounted in the snake-like robot through wireless module. This controller processes **O**_*s*_ by SVM and gets the predicting label. Then corresponding operation instructions are sent to the snake-like robot. Finally the snake-like robot executes the corresponding movement.

### 2.2. Snake-Like Robot

Snakes could do very well in the rough terrain like Mars, by going over and through broken ground and sand, and squeeze through tight spaces. Thus, great interest in the snake-like robot research has been generated. The European Space Agency is developing snake-like robots aiming at providing robot with more mobility during space exploratory activities. The snake-like robot applied in the mission of lunar exploration and Mars exploration will be helpful for the rover to travel over the complex rugged surface and narrow gaps on the ground.

During some missions where a wheeled rover collaborates with a snake-like robot, the wheeled rover can be used to travel long distances, while the snake robot could detach and reach places where the rover cannot reach. And if the rover gets stuck, the snake robot could conceivably be used to help pull it away.

Hirose has proposed the serpentine curve early in 1993 [11]. The curvature of the serpenoid curve is given by

(1) $$\begin{array}{c} \rho =-\alpha b\sin\left(\;bs\;\right), \end{array}$$

where *α* is amplitude angle (rad); *b* is constant of proportionality (rad/m); *s* is length of serpentine curve (m).

**Figure 3:**
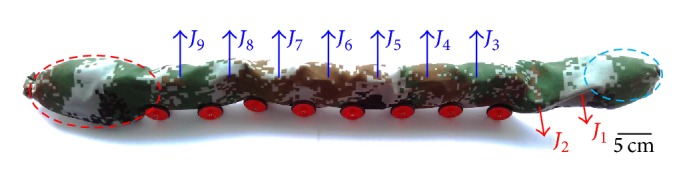
Structure of the snake-like robot.

**Figure 4:**
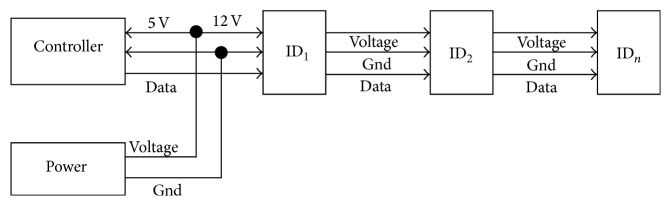
Physical connection.

The snake-like robot is composed of modular units, which are connected by active revolute joints, and the change of position between relative modules results in the movement of the robot. The flexible architecture of snake-like robot makes it hard to make a turning movement like other legged robots. To ensure the snake-like robot can achieve high efficiency in turning movement, Ye et al. proposed several methods for the turning motion of snake-like robot [12]. The snake-like robot used in this paper is shown in Figure 3 and made up of ten serial joints and each joint has one degree of freedom. A camera (the one encircled by the blue circle) is arranged on the head and a control module (the one encircled by the red circle) is fixed at the tail. Its physical connection is shown in Figure 4.

In the design of the communication system in a snake-like robot, a half-duplex asynchronous serial communication (8 bits, 1 stop, no parity) is utilized. Transmission speed is up to 1 Mbps. Link (physical) is TTL level multidrop (daisy chain type connector) considering minimizing physical cable.

**Figure 5:**
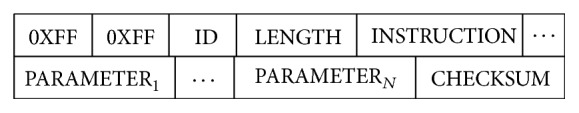
Snake-like robot communication protocol.

**Figure 6:**
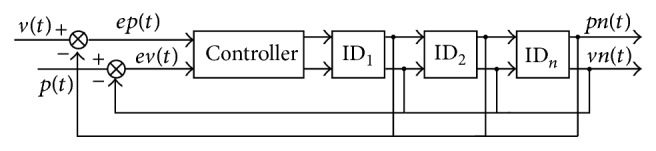
Control block structure.

The protocol of each modular unit communicating with the main controller is shown in Figure 5. Two 0XFF are the start code, ID is the number for the corresponding actuator, LENGTH is the length of the instruction, instruction is the instruction for the actuator to perform, PARAMETER is additional information needed to be sent other than the instruction, and the checksum is used to verify the signal. Distributed feedback compensation control is used as the control method. The specific control block diagram is shown in Figure 6.

## 3. Motion Recognition and Parameter Optimization

Machine learning based on data is an important aspect of modern intelligence technology. Statistics study begins with the observation of data to conclude a model, which is the base of the forecast for future data or the data cannot be observed. Traditional statistics study the asymptotic theory when the number of samples tends to infinity. Existing learning methods are mostly based on this assumption. But, in practical problems, the number of samples is often limited, so they usually have an unsatisfactory performance. Compared with the traditional statistics, Statistical Learning Theory (SLT) is a specialized theory, which systematically studies the relationship between experiences risk and actual risk for various types of sets of functions, namely, the generalization bounds [5]. Vapnik and Kotz began to dedicate themselves to researching this theory from the 1960s [13]. In the mid-90s, because of the development of Vapnik's theory and the lack of substantive progress in the theory of neural network learning methods, SLT began to receive more appreciation. SLT was based on a solid theory and provided a unified framework for solving the learning problem with the small samples. It incorporates many of the existing methods, expected to help solve many difficult problems, for example, the selection of neural network architecture and the local minima problem. Based on this theory, there is a new universal learning method; support vector machine (SVM), using geometry classification method to find the optimal hyperplane and get the maximum margin classifier, has shown a lot of superiority compared to the existing method [14, 15].

SVM is a more practical part of statistical theory, which was originally proposed by Vapnik et al. in 1992 to 1995 [14, 16–18]. It is currently still in the development stage. SVM is a structure of risk minimization strategies, which compromise the empirical risk and confidence interval to obtain the actual minimum risk [19]. A SVM approaches problems by searching for the Maximum Marginal Hyperplane (MMH) where a hyperplane has an equal distance from the hyperplane to both sides of its margin to ensure the hyperplane is more accurate at classifying future data tuples [20]. Compared with the new algorithms like Extreme Learning Machine (ELM) [21], SVM is committed to using less parameters to express a complex model; it still has its advantage in methodology and is more plausible.

**Figure 7:**
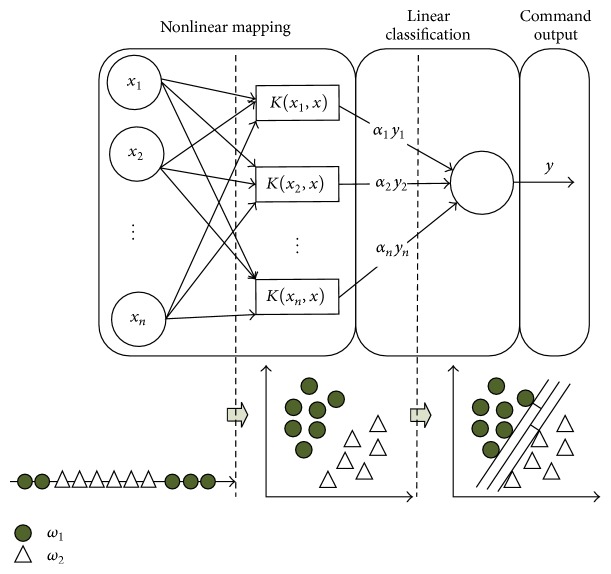
SVM nonlinear data processing principle.

SVM classifies linear data directly. When the data is linearly inseparable, it transforms the original data into a higher dimensional space by using a nonlinear mapping, and then searches for a linear separating hyperplane in the new space. Nonlinear data processing steps are shown in Figure 7.

There are several modes of SVM, which can be used for data classification, regression, and distribution estimation [22]. This paper uses the C-Support Vector Classification (C-SVC) [17, 23] to classify the data.

The distinguished hyperplane of the sample set **X** = {**x**_1_, **x**_1_,…, **x**_*N*_} can be shown by the formula

(2) $$\begin{array}{c} W^{T}x+w_{d+1}=0, \end{array}$$

where **W** is the weight vector and the direction of hyperplane. *d* is the dimension of the feature space. *w*_*d*+1_ is the offset of the hyperplane. During the course of looking for the best **W** ^*∗*^ to maximize the interval between the hyperplane and the closest sample, Lagrange multiplier method can be used to solve the problem of inequality constraint. The corresponding Lagrange function is

(3) LW,wn+1,λ=12WTW−∑k=1NλkykWTxk+wd+1−1,

where *λ*_*k*_ ≥ 0 and *k* = 1,2,…, *N* is the Lagrange coefficients to be determined.

To obtain a necessary condition for the extreme value in Lagrange function, the course of seeking the partial derivatives equaling zero of **W** and *w*_*d*+1_ is shown below:

(4) ∂∂WLW,wn+1,λW=W∗=W∗−∑k=1Nλkykxk=0,∂∂WLW,wn+1,λwd+1=wd+1∗=−∑k=1Nλkyk=0.

Namely,

(5) W∗=∑k=1Nλkykxk,∑k=1Nλkyk=0.

Convert it to the dual form:

(6) $$\begin{array}{c} L_{D}\left(\;\lambda \;\right)=-\frac{1}{2}\overset{N}{\underset{i=1}{\sum }}\;\overset{N}{\underset{j=1}{\sum }}\lambda_{i}\lambda_{j}y_{i}y_{j}x_{i}^{T}x_{j}+\overset{N}{\underset{k=1}{\sum }}\lambda_{k}. \end{array}$$

To ensure distinguished hyperplane has the smallest risk of classification,

(7) Maximise LDλ=Maximise∑k=1Nλk−12∑i=1N ∑j=1NλiλjyiyjxiTxjSubject  to λk≥0,  k=1,2,…,N, ∑k=1Nλkyk=0.

The function showed above is the simple quadratic programming problem, which has standard solving algorithm. Once the problem is solved under the condition of *λ*_*k*_ ≥ 0, *k* = 1,2,…, *N*, the optimal weight vector **W** ^*∗*^ will be got based on the formula shown in (5). Solutions meeting the requirements are called support vector.

When it comes to nonlinear classification, the data is usually mapped to a high-dimensional linear space by the kernel function in Figure 7. In this way the linearly inseparable data can be converted into linear separable data in a high-dimensional space. Three kinds of kernel functions are commonly used, namely, polynomial kernel of degree *h*, Gaussian radial basis function kernel, and Sigmoid kernel. Three kernel functions are as follows.

Polynomial kernel of degree *h* is

(8) $$\begin{array}{c} K\left(\;x_{i},x_{j}\;\right)={\left(\;x_{i}\cdot x_{j}+1\;\right)}^{h}. \end{array}$$

Gaussian radial basis function kernel is

(9) $$\begin{array}{c} K\left(\;x_{i},x_{j}\;\right)=e^{-{\left\|x_{i}\cdot x_{j}-1\right\|}^{2}/2\sigma^{2}}. \end{array}$$

Sigmoid kernel is

(10) $$\begin{array}{c} K\left(\;x_{i},x_{j}\;\right)=\tanh\left(\;\kappa x_{i}\cdot x_{j}-\delta \;\right). \end{array}$$

There are no golden rules for determining which admissible kernel will result in the most accurate result in SVM. In practice, the kernel chosen does not generally make a large difference in the resulting accuracy. SVM training always finds a global solution, unlike neural networks, such as backpropagation, where many local minima usually exist.

For the using of SVM, although the choosing of kernel generally does not make a large difference in result accuracy, when a kernel is chosen, there are still a number of parameters that should be optimized. In this paper, after selecting the Gaussian radial basis function, there are two parameters *c* and *g* that need to be optimized, where *c* is the penalty coefficient that means error tolerance; the higher the value is, the smaller the error can be tolerated. Parameter *g* determines the distribution of data after mapping to the new feature space.

There is no best way to select the SVM parameters. The most common way is to let *c* and *g* be within a certain range. In this paper, cross-validation method based on grid-search was used for the parameter optimization. Cross-validation is one of the more classic solutions [22]. The algorithm is conducted according to a basic idea that in the inner loop of cross-validation, once the recognition rate for the first time appears to be a local maximum, the parameter values are recorded and the inner loop ends. Finally, estimate the optimal parameters by calculating the arithmetic mean of the entire local maximum.

PSO is a new Evolutionary Algorithm (EA) developed in recent years [24]. The particle swarm is more than just a collection of particles. A particle by itself has almost no power to solve any problem. Progress occurs only when the particles interact. Particle swarm follows the optimal particle to search the solution space; each particle obtains a search direction and speed in next loop by comparing with the individual optimum value and global optimum value respectively with random perturbations distributed uniformly in a certain range. Compared with other EAs, the advantages of PSO are being simple, being easy to achieve, and few parameters to be adjusted. Using PSO with appropriate parameters can significantly improve the accuracy of SVM [25–28]. The formulas to update the primitive velocity and location are shown as follows:

(11) v→i⟵ωv→i+U→0,ϕ1⊗p→i−x→i+U→0,ϕ2⊗p→g−x→i,x→i⟵x→i+v→i,

where ${\vec{x}}_{i}$ is the current location; ${\vec{p}}_{i}$ is the previous personal best position; ${\vec{p}}_{g}$ is the previous global best position; ${\vec{v}}_{i}$ is velocity and *ω* is inertia weight; $\vec{U}(0,\phi_{i})$ represents a vector of random numbers uniformly distributed in [0, *φ*_*i*_] which is randomly generated at each iteration and for each particle; ⊗ is componentwise multiplication

In the original version of PSO, velocity of each particle is limited to [−*V*_max_, +*V*_max_].

**Figure 8:**
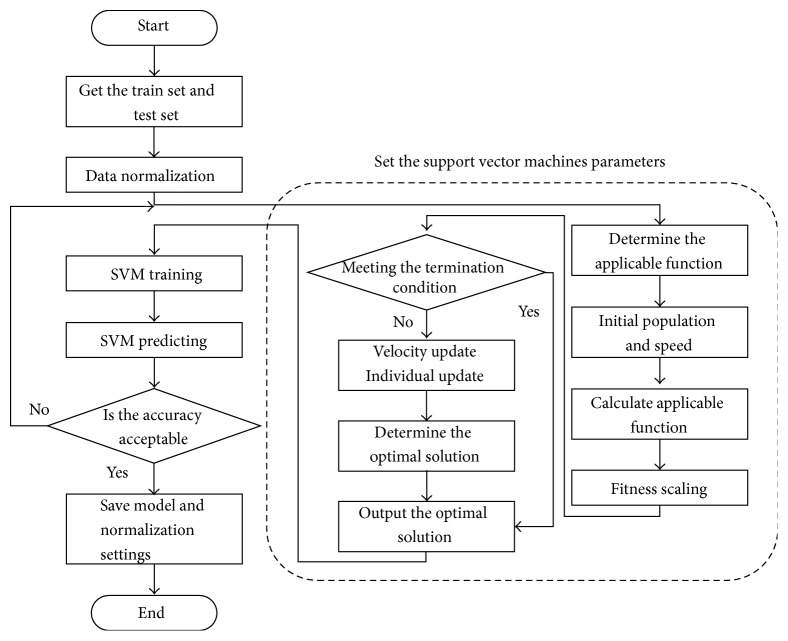
Flow diagram of the overall program.

The program flow using the SVM, whose parameters were chosen by the PSO to obtain a classification model, is shown in Figure 8.

## 4. Experiment

In this paper, we focus on the planet surface EVA, where the autonomous robots need assistance on path planning, mission guidance, and so forth. In the process of classification of HRI instructions, the above learning method, SVM, with a small number of learning samples is used to classify the instructions. For the SVM parameter optimization, PSO algorithm was used to optimize SVM parameters *c* and *g* by the way of cross-validation. The found optimal parameters will be used to find the best SVM model. The software package LIBSVM we used was developed in [22].

In order to verify the accuracy and robustness of the proposed method, two experiments are conducted. First, the proposed methods are evaluated on 16 hand gestures selected from 36 hand gestures in the ASL. Second, the hand recognition algorithm has been integrated into a snake-like robot, and validation is then made with a space suit.

### 4.1. Hand Gesture Recognition

ASL has 36 hand gestures, 26 letters, and 6000 words. Although most of the ASL alphabet letters depend on finger bending, some of them also depend on hand orientation and two of them are dynamic. There are some similarities between *g* and *q*, *h* and *u*, and *k* and *p*. These couples have basically the same hand shape, but their hand orientation differs from the others. There are hand shape similarities between *i* and *j* and *x* and *z*, but *j* and *z* are dynamic characters.

**Figure 9:**
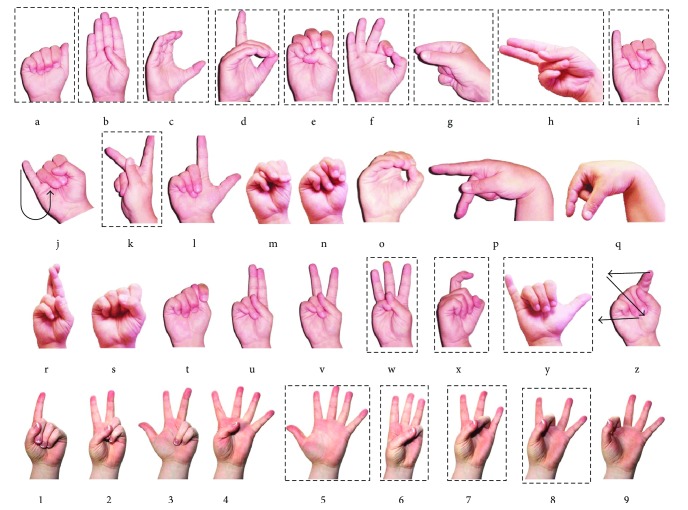
16 kinds of gestures in ASL.

**Figure 10:**
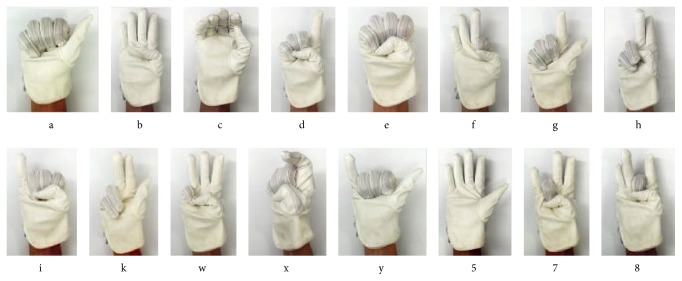
Corresponding gestures in experiment.

In this paper, we selected 16 in 36 of ASL shown in Figure 9 for the classification and identification experiment; corresponding gestures in the experiment are shown in Figure 10. For each gesture, we collected 15 sets of data, from which we use 10 for training and other 5 for the testing. The test data is normalized before testing the accuracy. In addition, we collected 5 new sets of hand shapes for each gesture to test the trained model.

For a more detailed analysis on the effect of using PSO for SVM cross-validation, we calculated the average Cross-Validation Accuracy (CVA) of the SVM cross-validation for different parameters of PSO, as shown in Tables 2 and 3 (each group has 6 experiments). At first, we fixed the maximum generation as 5 and adjusted the size of PSO population shown in Table 2. The parameter of PSO is the maximum generation or the size of PSO population. Obviously, when the size of PSO population was equal to 5, the CVA reached its maximum. With the population as 5 and the maximum generation, the results shown in Table 3 indicated that when the maximum generation is equal to 5, the CVA reached its maximum.

From Tables 2 and 3, we can also know that using PSO with appropriate parameters can significantly improve the accuracy of SVM in the process of cross-validation. Compared with the results of gesture recognition using ELM in [29], few parameters were used in this paper, and SVM had more stable results than the Extreme Learning Machine.

As we can see, the classification accuracy can always reach 100% except the size of PSO population that is too small. It demonstrates that the method in this paper has a high accuracy and strong robustness.

### 4.2. Snake-Like Robot Remote Control with Hand Gestures

**Figure 11:**
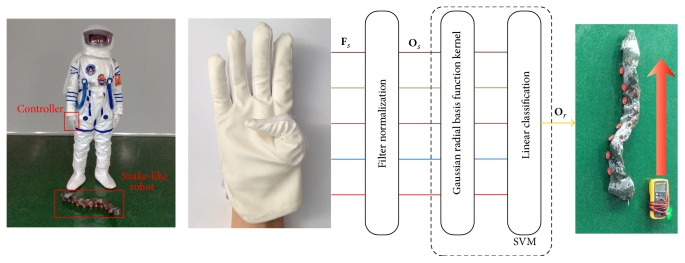
Experimental system.

A snake-like robot plays a powerful role in space exploratory activities. In this paper, a snake-like robot motion control was employed of testing the accuracy, stability, and robustness of the proposed approach. We modeled the environment of astronauts on other planets, embedded the controller in the glove, and controlled the movement of the snake-like robot. Overall structure of the experiment is shown in the left of Figure 11 and a schematic diagram of control signal flow shown in the right.

**Figure 12:**
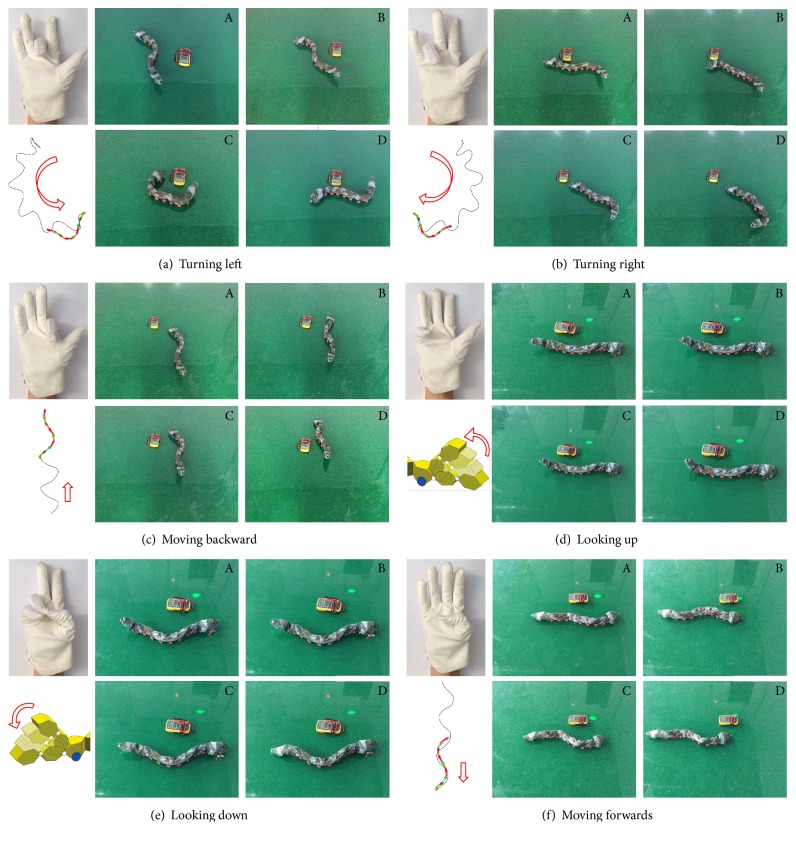
Snake-like robot gesture control experiments.

The hand gestures have been integrated into the glove-robot control system. Various motions have been identified for the snake-like robot, such as turning left/right and moving forward/backward. It demonstrates that the proposed system including the hardware and software is effective and robust. It is a good prototype for the HRI used in the space exploration. The corresponding hand gestures, robot movements, and simulated motion tracks are shown in Figure 12, respectively.

## 5. Conclusion and Future Works

This paper proposed a gesture-type user control system for the space exploration based on the actual application environment for the purpose of utility and stability. In this study, bending sensors were integrated with a space suit to control a snake-like robot, which was designed for the space exploration. SVM was used as the gesture signal pattern recognizer, and PSO algorithm was used for optimizing the parameters of SVM. The system classified the action sequence and ensured the accuracy and real-time performance of the control process. The experimental results showed that this system was effective with a high accuracy, reliability, and robustness.

In the future, the system will be improved with a series of command functions so that astronauts can interrupt robot's operations whenever necessary to provide guidance and assistance for the mission. Simultaneously, the collaboration between the astronaut and the robot will be strengthened and the interactions will be more precise and concise with advanced nonlinear methods [30, 31]. Finally, the HRI system will be further improved with a natural and friendly interface so that nontechnical astronauts can also have a barrier-free communication with robots.

## Figures and Tables

**Table 1:** Main parameters of the device.

Items	Properties
Bend sensors	Temperature range: −35°C~+80°C; resistance tolerance: ±30%

Stm32 controller	Cores: Cortex-M3 32-bit RISC, 512 K Flash, 64 K RAM; operating frequency: 72 MHz, 1.25 DMIPS/MHz

NRF24L01	Transmission distance: 150 m; digital interface (SPI) speed: 0~10 Mbps; on the air data rate 1 or 2 Mbps

Servos	Power supply range: 7~10 V; operating temperature: −5°C~+85°C; communication speed: 7343 bps~1 Mbps

**Table 2:** Average CVA when the maximum generation is fixed.

Parameter of PSO	CVA	Classification accuracy
5∖2	55.1968%	64.79%
5∖3	77.1160%	100%
5∖4	77.2569%	100%
5∖5	81.3294%	100%
5∖6	78.9453%	100%
5∖7	77.5563%	100%

**Table 3:** Average CVA when the size of PSO population is fixed.

Parameter of PSO	CVA	Classification accuracy
2∖5	74.1435%	100%
3∖5	76.9485%	100%
4∖5	76.7045%	100%
5∖5	81.3294%	100%
6∖5	80.3364%	100%
7∖5	80.2778%	100%
